# 1,1′-(Piperazine-1,4-di­yl)dipropan-2-ol

**DOI:** 10.1107/S1600536811023397

**Published:** 2011-06-22

**Authors:** Murat Türkyılmaz, Yakup Baran, Namık Özdemir

**Affiliations:** a Department of Chemistry, Faculty of Science, Trakya University, 22030-Edirne, Turkey; b Department of Chemistry, Faculty of Arts and Sciences, Çanakkale Onsekiz Mart University, 17020-Çanakkale, Turkey; c Department of Physics, Faculty of Arts and Sciences, Ondokuz Mayıs University, 55139-Samsun, Turkey

## Abstract

The asymmetric unit of the crystal contains one-fourth of the title compound, C_10_H_22_N_2_O_2_, with the centre of the piperazine ring located at a site of 2/*m* symmetry. The piperazine ring adopts a chair conformation. The methine and methylene C atoms of the 2-hydroxypropyl groups show symmetry-imposed disorder over two equally occupied and mutually exclusive sets of positions. Only intra­molecular O—H⋯N contacts are observed.

## Related literature

For the biological properties of piperazine compounds, see: Foroumadi *et al.* (2007 ▶); Upadhayaya *et al.* (2004 ▶); Chen *et al.* (2006 ▶); Cunico *et al.* (2009 ▶); Smits *et al.* (2008 ▶); Penjišević *et al.* (2007 ▶); Becker *et al.* (2006 ▶). For hydrogen-bond graph-set motifs, see: Bernstein *et al.* (1995 ▶). For ring puckering parameters, see: Cremer & Pople (1975 ▶).
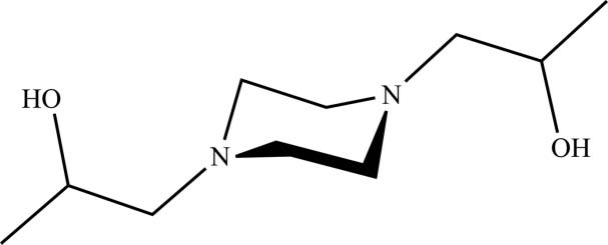


## Experimental

### 

#### Crystal data

C_10_H_22_N_2_O_2_
*M* *_r_* = 202.30
Monoclinic, 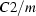

*a* = 13.838 (10) Å
*b* = 7.791 (5) Å
*c* = 5.543 (4) Å
β = 97.26 (3)°
*V* = 592.8 (7) Å^3^
*Z* = 2
Mo *K*α radiation
μ = 0.08 mm^−1^
*T* = 273 K
0.40 × 0.25 × 0.10 mm

#### Data collection

Rigaku R-AXIS RAPID diffractometer
2493 measured reflections
604 independent reflections
441 reflections with *I* > 2σ(*I*)
*R*_int_ = 0.139

#### Refinement

*R*[*F* ^2^ > 2σ(*F* ^2^)] = 0.052
*wR*(*F* ^2^) = 0.119
*S* = 1.05
604 reflections
46 parameters
14 restraints
H-atom parameters constrained
Δρ_max_ = 0.28 e Å^−3^
Δρ_min_ = −0.34 e Å^−3^

### 

Data collection: *PROCESS-AUTO* (Rigaku, 1998 ▶); cell refinement: *PROCESS-AUTO*; data reduction: *CrystalStructure* (Rigaku/MSC, 2004 ▶); program(s) used to solve structure: *SHELXS97* (Sheldrick, 2008 ▶); program(s) used to refine structure: *SHELXL97* (Sheldrick, 2008 ▶); molecular graphics: *ORTEP-3 for Windows* (Farrugia, 1997 ▶); software used to prepare material for publication: *WinGX* (Farrugia, 1999 ▶) and *PLATON* (Spek, 2009 ▶).

## Supplementary Material

Crystal structure: contains datablock(s) I, global. DOI: 10.1107/S1600536811023397/fy2011sup1.cif

Structure factors: contains datablock(s) I. DOI: 10.1107/S1600536811023397/fy2011Isup2.hkl

Supplementary material file. DOI: 10.1107/S1600536811023397/fy2011Isup3.cml

Additional supplementary materials:  crystallographic information; 3D view; checkCIF report

## Figures and Tables

**Table 1:** Hydrogen-bond geometry (Å, °)

*D*—H⋯*A*	*D*—H	H⋯*A*	*D*⋯*A*	*D*—H⋯*A*
O1—H1⋯N1	0.82	2.22	2.696 (3)	117

**Fig. 1.**
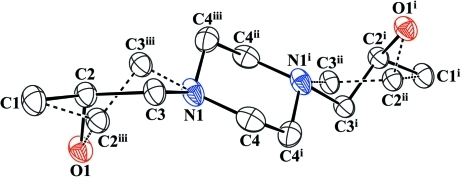
ORTEP-3 drawing of the title compound, showing 30% probability displacement ellipsoids and the atomic numbering scheme. For the sake of clarity, H atoms have been omitted. One of the disorder components is drawn with dashed bonds. [Symmetry codes: (i) 1 - x, y, 1 - z; (ii) 1 - x, 1 - y, 1 - z; (iii) x, 1 - y, z.]

## supplementary crystallographic information

### Comment

Piperazine-based research has attracted considerable attention in recent years. A broad range of compounds displaying antibacterial (Foroumadi *et al.*, 2007), antifungal (Upadhayaya *et al.*, 2004), anticancer (Chen *et al.*, 2006), antiparasitic (Cunico *et al.*, 2009), antihistamin (Smits *et al.*, 2008), psychotolytic (Penjišević *et al.*, 2007), and antidepressive activities (Becker *et al.*, 2006) have been found to contain this versatile core. In view of these important properties, we have undertaken the X-ray diffraction study of the title compound.

The structure of the title compound is shown in Fig. 1. The structure contains one central piperazine ring (N1/C4/C4^i^/N1^i^/C4^ii^/C4^iii^) with two propanol moieties substituted at the two N atoms of the piperazine ring. The centre of the ring located at a site of 2/*m* symmetry. The N, O and methyl C atoms are located on the mirror plane, while atoms C2 and C3 show symmetry-imposed disorder.

The interatomic distances and angles in the compound show no anomalies. The piperazine ring adopts a chair conformation, as is evident from the puckering parameters (Cremer & Pople, 1975): Q_T_ = 1.0333 (10) Å, q_2_ = 0.8812 (9) Å, q_3_ = 0.5396 (6) Å, θ = 58.52 (2)° and φ_2_ = 30.00 (5)° for the atom sequence N1/C4/C4^i^/N1^i^/C4^ii^/C4^iii^. Atoms N1 and N1^i^ are on opposite sides of the C4/C4^i^/C4^ii^/C4^iii^ plane and are both displaced from it by 0.2424 (30) Å.

The molecular structure of the title compound contains two intramolecular O—H···N contacts, which form a five-membered ring with graph-set descriptor *S*(5) (Bernstein *et al.*, 1995). No intermolecular hydrogen bonds are observed in the crystal structure. Van der Waals forces stabilize the packing.

### Experimental

Piperazine (1.50 g, 17.40 mmol) was dissolved in 50 ml argon saturated methanol. Methanol solution of 2.88 g (50.00 mmol) propylene oxide was added to the piperazine solution at room temperature. The solution was left under magnetic stirrer for 24 h. The solution volume was reduced by rotary evaporator and the oily product was left for crystallization.

### Refinement

H atoms were positioned geometrically and treated using a riding model, fixing the bond lengths at 0.82, 0.93, 0.97 and 0.96 Å for OH, CH, CH_2_ and CH_3_ groups, respectively. The isotropic displacement parameters of the H atoms were constrained at 1.2 *U*_eq_ of their parent atom (1.5 *U*_eq_ for methyl and OH groups). Atoms C2 and C3 showed symmetry-imposed disorder and were refined anisotropically using ADP restraints (SIMU and DELU) and half occupancy.

### Crystal data

**Table:**

C_10_H_22_N_2_O_2_	*F*(000) = 224
*M**_r_* = 202.30	*D*_x_ = 1.133 Mg m^−^^3^
Monoclinic, *C*2/*m*	Mo *K*α radiation, λ = 0.71073 Å
Hall symbol: -C 2y	Cell parameters from 3092 reflections
*a* = 13.838 (10) Å	θ = 3.0–30.0°
*b* = 7.791 (5) Å	µ = 0.08 mm^−^^1^
*c* = 5.543 (4) Å	*T* = 273 K
β = 97.26 (3)°	Prism, colourless
*V* = 592.8 (7) Å^3^	0.40 × 0.25 × 0.10 mm
*Z* = 2	

### Data collection

**Table:**

Rigaku R-AXIS RAPID diffractometer	441 reflections with *I* > 2σ(*I*)
Radiation source: fine-focus sealed tube	*R*_int_ = 0.139
graphite	θ_max_ = 25.5°, θ_min_ = 3.0°
Detector resolution: 10.00 pixels mm^-1^	*h* = −16→16
ω scans	*k* = −9→9
2493 measured reflections	*l* = −5→6
604 independent reflections	

### Refinement

**Table:**

Refinement on *F*^2^	Primary atom site location: structure-invariant direct methods
Least-squares matrix: full	Secondary atom site location: difference Fourier map
*R*[*F*^2^ > 2σ(*F*^2^)] = 0.052	Hydrogen site location: inferred from neighbouring sites
*wR*(*F*^2^) = 0.119	H-atom parameters constrained
*S* = 1.05	*w* = 1/[σ^2^(*F*_o_^2^) + (0.0375*P*)^2^ + 0.2898*P*] where *P* = (*F*_o_^2^ + 2*F*_c_^2^)/3
604 reflections	(Δ/σ)_max_ < 0.001
46 parameters	Δρ_max_ = 0.28 e Å^−^^3^
14 restraints	Δρ_min_ = −0.34 e Å^−^^3^

### Special details

**Table:**

Geometry. All e.s.d.'s (except the e.s.d. in the dihedral angle between two l.s. planes) are estimated using the full covariance matrix. The cell e.s.d.'s are taken into account individually in the estimation of e.s.d.'s in distances, angles and torsion angles; correlations between e.s.d.'s in cell parameters are only used when they are defined by crystal symmetry. An approximate (isotropic) treatment of cell e.s.d.'s is used for estimating e.s.d.'s involving l.s. planes.
Refinement. Refinement of *F*^2^ against ALL reflections. The weighted *R*-factor *wR* and goodness of fit *S* are based on *F*^2^, conventional *R*-factors *R* are based on *F*, with *F* set to zero for negative *F*^2^. The threshold expression of *F*^2^ > σ(*F*^2^) is used only for calculating *R*-factors(gt) *etc*. and is not relevant to the choice of reflections for refinement. *R*-factors based on *F*^2^ are statistically about twice as large as those based on *F*, and *R*- factors based on ALL data will be even larger.

### Fractional atomic coordinates and isotropic or equivalent isotropic displacement parameters (Å^2^)

**Table:**

	*x*	*y*	*z*	*U*_iso_*/*U*_eq_	Occ. (<1)
O1	0.78313 (12)	0.5000	0.4674 (3)	0.0706 (7)	
H1	0.7297	0.5197	0.3907	0.106*	0.50
N1	0.60084 (15)	0.5000	0.5912 (4)	0.0715 (7)	
C1	0.86326 (18)	0.5000	0.8733 (5)	0.0754 (9)	
H1A	0.9169	0.4390	0.8197	0.113*	0.50
H1B	0.8573	0.4690	1.0384	0.113*	0.50
H1C	0.8746	0.6213	0.8640	0.113*	0.50
C2	0.7712 (2)	0.4545 (5)	0.7137 (5)	0.0603 (12)	0.50
H2	0.7597	0.3307	0.7233	0.072*	0.50
C3	0.6841 (2)	0.5495 (5)	0.7803 (6)	0.0609 (11)	0.50
H3A	0.6952	0.6724	0.7782	0.073*	0.50
H3B	0.6705	0.5166	0.9413	0.073*	0.50
C4	0.54111 (14)	0.6506 (3)	0.6021 (3)	0.0751 (7)	
H4A	0.5806	0.7526	0.5917	0.090*	
H4B	0.5150	0.6533	0.7565	0.090*	

### Atomic displacement parameters (Å^2^)

**Table:**

	*U*^11^	*U*^22^	*U*^33^	*U*^12^	*U*^13^	*U*^23^
O1	0.0609 (11)	0.0975 (15)	0.0541 (11)	0.000	0.0103 (8)	0.000
N1	0.0463 (11)	0.118 (2)	0.0498 (12)	0.000	0.0031 (9)	0.000
C1	0.0527 (15)	0.103 (2)	0.0677 (17)	0.000	−0.0030 (13)	0.000
C2	0.0519 (15)	0.075 (3)	0.0533 (16)	0.0024 (15)	0.0027 (13)	−0.0010 (15)
C3	0.0483 (14)	0.084 (3)	0.0490 (14)	0.0021 (14)	0.0016 (12)	−0.0047 (14)
C4	0.0807 (13)	0.0883 (15)	0.0563 (11)	−0.0195 (11)	0.0084 (9)	−0.0038 (11)

### Geometric parameters (Å, °)

**Table:**

O1—C2^i^	1.440 (4)	C1—H1B	0.9600
O1—C2	1.440 (4)	C1—H1C	0.9600
O1—H1	0.8200	C2—C3	1.499 (4)
N1—C4^i^	1.441 (3)	C2—H2	0.9800
N1—C4	1.441 (3)	C3—H3A	0.9700
N1—C3	1.507 (3)	C3—H3B	0.9700
N1—C3^i^	1.507 (3)	C4—C4^ii^	1.500 (4)
C1—C2	1.499 (4)	C4—H4A	0.9700
C1—C2^i^	1.499 (4)	C4—H4B	0.9700
C1—H1A	0.9600		
			
C2^i^—O1—H1	103.9	O1—C2—C3	107.7 (2)
C2—O1—H1	109.5	C1—C2—C3	112.8 (3)
C4^i^—N1—C4	109.0 (2)	O1—C2—H2	109.4
C4^i^—N1—C3	124.8 (2)	C1—C2—H2	109.4
C4—N1—C3	98.90 (17)	C3—C2—H2	109.4
C4^i^—N1—C3^i^	98.90 (17)	C2—C3—N1	105.7 (2)
C4—N1—C3^i^	124.8 (2)	C2—C3—H3A	110.6
C2—C1—H1A	109.5	N1—C3—H3A	110.6
C2^i^—C1—H1A	124.6	C2—C3—H3B	110.6
C2—C1—H1B	109.5	N1—C3—H3B	110.6
C2^i^—C1—H1B	116.9	H3A—C3—H3B	108.7
H1A—C1—H1B	109.5	N1—C4—C4^ii^	110.64 (15)
C2—C1—H1C	109.5	N1—C4—H4A	109.5
C2^i^—C1—H1C	82.4	C4^ii^—C4—H4A	109.5
H1A—C1—H1C	109.5	N1—C4—H4B	109.5
H1B—C1—H1C	109.5	C4^ii^—C4—H4B	109.5
O1—C2—C1	108.1 (2)	H4A—C4—H4B	108.1
			
C2^i^—O1—C2—C1	−70.1 (2)	C4^i^—N1—C3—C2	85.4 (3)
C2^i^—O1—C2—C3	52.0 (2)	C4—N1—C3—C2	−153.8 (2)
C2^i^—C1—C2—O1	69.7 (2)	C3^i^—N1—C3—C2	52.8 (3)
C2^i^—C1—C2—C3	−49.2 (3)	C4^i^—N1—C4—C4^ii^	−58.1 (3)
O1—C2—C3—N1	56.3 (3)	C3—N1—C4—C4^ii^	170.2 (2)
C1—C2—C3—N1	175.4 (2)	C3^i^—N1—C4—C4^ii^	−174.2 (2)

Symmetry codes: (i) *x*, −*y*+1, *z*; (ii) −*x*+1, *y*, −*z*+1.

### Hydrogen-bond geometry (Å, °)

**Table:**

*D*—H···*A*	*D*—H	H···*A*	*D*···*A*	*D*—H···*A*
O1—H1···N1	0.82	2.22	2.696 (3)	117
